# Association Between Salivary Sirtuin-1 Levels and Periodontitis

**DOI:** 10.3390/jcm15041485

**Published:** 2026-02-13

**Authors:** Leonardo Lorente, Esther Hernández Marrero, Pedro Abreu González, Angel Daniel Lorente Martín, Marina Lorente Martín, María José Marrero González, Carmen Hernández Marrero, Olga Hernández Marrero, Alejandro Jiménez, Cándido Manuel Hernández Padilla

**Affiliations:** 1 Intensive Care Unit, Hospital Universitario de Canarias, Ofra s/n, 38320 San Cristóbal de La Laguna, Tenerife, Spain; 2 Clínica Dental Cándido, Plaza San Cristóbal 35, 38204 San Cristóbal de La Laguna, Tenerife, Spain; esther@clinicadentalcandido.com (E.H.M.); mariajose@clinicadentalcandido.com (M.J.M.G.); carmen@clinicadentalcandido.com (C.H.M.); olga@clinicadentalcandido.com (O.H.M.); candido@clinicadentalcandido.com (C.M.H.P.); 3 Unit of Physiology, Department of Basic Medical Sciences, Faculty of Medicine, University of La Laguna, Ofra, s/n, 38320 San Cristóbal de La Laguna, Tenerife, Spain; pabreu@ull.edu.es; 4 Department of Odontology, Faculty of Medicine, CEU San Pablo University, Avenida Montepríncipe s/n, 28660 Boadilla del Monte, Madrid, Spain; daniellorentemartin@gmail.com; 5 Department of Nursing, Faculty of Nursing and Physiotherapy, Salus Infirmorum-Universidad Pontificia de Salamanca, Calle de Gaztambide 12, 28015 Madrid, Madrid, Spain; marinalorente17@gmail.com; 6 Research Unit, Hospital Universitario de Canarias, Ofra, s/n, 38320 San Cristóbal de La Laguna, Tenerife, Spain; ajimenezsosa@gmail.com

**Keywords:** sirtuin-1, salivary, periodontitis, oxidation

## Abstract

**Background:** Sirtuin-1 (SIRT-1) is one enzyme that has anti-oxidative, anti-inflammation and anti-apoptosis effects, and it is involved in regulating aging and in different age-associated disorders. Salivary SIRT-1 concentrations in periodontitis patients have been scarcely studied and only in studies with small sample sizes (the highest with 83 subjects). There were no differences in salivary SIRT-1 concentrations between periodontitis patients and periodontally healthy subjects in any of those studies. The aims of our study were to compare salivary SIRT-1 concentrations in subjects with periodontitis and those without it in a study with a larger sample size to determine whether there exists an association between salivary SIRT-1 concentrations and the presence of periodontitis and to analyze the potential capability of salivary SIRT-1 concentrations for periodontitis diagnosis. **Material and Methods:** In this cross-sectional study, salivary SIRT-1 concentrations were measured in subjects with and without periodontitis. To determine that variables were associated with periodontitis, a multivariate logistic regression analysis was carried out. To determine the capability of salivary SIRT-1 concentrations to diagnose periodontitis, a receiver operating characteristic (ROC) analysis was carried out. **Results:** We included 144 subjects (85 without periodontitis and 59 with periodontitis). Salivary SIRT-1 concentrations < 1.2 ng/mL were associated independently with periodontitis (OR = 2.84; 95% CI = 1.075–7.506; *p* = 0.04) in the regression analysis. The salivary SIRT-1 concentration showed an area under curve of 74% (95% CI = 66–86%; *p* < 0.001) for periodontitis diagnosis in the ROC analysis. **Conclusions:** Our study had the highest sample size reporting salivary SIRT-1 concentrations in patients with periodontitis. We found that low salivary SIRT-1 concentrations could be associated with the presence of periodontitis. In addition, they could play a role in periodontitis diagnosis as an adjunct to other markers given that their diagnostic capability is not high. However, given the limitations of our study, further investigations are necessary to confirm these preliminary findings.

## 1. Introduction

Periodontitis is a chronic disease in which chronic inflammation of the periodontium could produce loss of bone and tooth. This chronic disease has a high prevalence and can affect up to 90% of the population [1], and its severe form can affect up to 15% of the population [2].

Periodontitis is characterized by gingival inflammation related to microbial agents, which leads to loss of periodontal attachment and, finally, tooth loss [3,4]. The formation of a bacterial biofilm initiates gingival inflammation, erythema, swelling, bleeding, periodontal pockets, recessions of the gingival margins, loss of periodontal ligament fibers and potential loss of alveolar bone. Ultimately, tooth mobility, migration, tilting and tooth loss can occur. Various pathophysiological pathways are activated in periodontitis such as oxidative stress [5], inflammation [6] and programmed cell death [7].

Several risk factors for periodontitis have been reported, including tea, tobacco, alcohol, and coffee, immunosuppression, oral cancer, dental hygiene, consumption of drugs [4], age [8], obesity [9], rheumatoid arthritis [10], arterial hypertension [11], diabetes mellitus [12], systemic lupus erythematosus [13], sex [14], statins [15], metformin [16], socioeconomic status and diet [17].

Sirtuins or Silent Information Regulators (SIRTs) are a family of enzymes that are involved in the modulation of oxidative stress responses, inflammation and apoptosis [18,19,20,21,22,23,24,25,26,27,28]. SIRTs have been extensively studied in regulating aging (promoting longevity) [18,19,20,21] and in different age-associated diseases, such as liver, kidney, cardiovascular, brain, bowel and lung diseases; diabetes mellitus; and cancer [22,23,24,25,26,27,28]. In addition, sirtuins also have a role in different oral diseases such as periodontitis and apical periodontitis and in others such as oral cancer, pulpitis, dental fluorosis, oral herpesvirus infections or oral candidiasis [29].

The SIRT family consists of seven members (SIRT-1 to SIRT-7), and each member has different and inclusive contrary effects on some diseases [18,19,20,21,22,23,24,25,26,27,28,29]. SIRT-1 is the most common SIRT studied. The activation of SIRT-1 depends on the coenzyme nicotinamide adenine dinucleotide (NAD). NAD has two forms, NAD^+^ (which is the oxidized form) and NADH (which is the form reduced form). NADH oxidase plays an important role in the production of reactive oxygen species (ROS) by catalyzing the oxidation of NADH to NAD^+^ and transferring an electron to oxygen, resulting in the formation of NAD^+^ and the superoxide radical (NADH + O_2_ = NAD^+^ + O_2_^−^). The action of SIRT-1 depends on NAD^+^, and the increase in the NAD^+^/NADH ratio activates SIRT-1.

SIRTs have different enzymatic activities, but deacetylation is the predominant enzymatic process (removing acetyl groups from different proteins, thus reducing the amount of various acetylated proteins); however, SIRTs have other enzymatic activities such as depropanediylation, desuccinylation, adenosine diphosphate ribosylation, demyristoylation and deglutarylation.

SIRT-1 has antioxidative, anti-inflammatory and anti-apoptotic effects since it can regulate the activation of different transcriptional factors such as nuclear factor erythroid 2-related factor 2 (Nrf2), forkhead box protein O3 (FOXO3a), nuclear factor-kappa B (NF-κB), and transcription factor p53.

SIRT-1 can activate FOXO3a by its deacetylation, leading to elevated expression of antioxidant enzymes such as manganese superoxide dismutase (MnSOD) and catalase.

In addition, SIRT-1 can activate Nrf2 by its deacetylation, leading to upregulated expression of antioxidant enzymes such as superoxide dismutase (SOD), NAD(P)H quinone dehydrogenase 1 (NQO1) and heme oxygenase 1 (HO-1).

Additionally, SIRT-1 can inhibit the activity of NF-κB by its deacetylation, leading to decreased expression of pro-inflammatory cytokines such as interleukin (IL)-1β, IL-6, IL-8, and tumor necrosis factor α (TNF-α).

In addition, SIRT-1 has anti-apoptotic effects due to the inhibition of protein p53 by its deacetylation, leading to decreased expression of pro-apoptotic proteins (Bax and Bim), and increased expression of the anti-apoptotic Bcl-2 protein.

Therefore, due to its anti-oxidative, anti-inflammation and anti-apoptosis effects, SIRT-1 plays an important role in aging and in the pathophysiology of various diseases. Different agents with an activating or inhibiting effect on SIRT-1 have been explored [30,31,32,33,34,35]. Thus, the administration of SIRT-1 activators could be beneficial in age-associated diseases. SIRT-1 inhibitors may have therapeutic potential as anticancer agents by promoting apoptosis and tumor suppression. Nevertheless, each member of the SIRT family has distinct functions and contributes differently to aging and age-associated diseases [18,19,20,21,22,23,24,25,26,27,28,29]. For example, SIRT-1 inhibitors and SIRT-6 activators could be used to treat cancer (due to reduced tumorigenesis, cancer progression and antitumoral drug resistance). On the other hand, SIRT-1 activators and SIRT-6 inhibitors could be used for diabetes mellitus treatment.

SIRTs in periodontitis have been scarcely studied, and different biological samples (serum, saliva, and gingival tissue samples) have been used [36,37,38,39,40,41,42,43,44,45]. Regarding salivary SIRT-1 levels, no differences were found between periodontitis patients and periodontally healthy subjects in any study [36,37,38]; in addition, these studies had small sample sizes (the largest included 83 subjects in the study by Kluknavská [36]). Regarding serum SIRT-1 levels, no differences were found between patients with periodontitis and periodontally healthy subjects [39]; higher serum SIRT-1 levels were reported in periodontitis patients compared to periodontally healthy subjects [40], and lower serum SIRT-1 levels were found in smokers with periodontitis compared to periodontally healthy subjects [41]. Regarding SIRT-1 expression in gingival tissue, both higher [42] and lower expression levels of SIRT-1 were reported in periodontitis patients compared to periodontally healthy individuals [43]. Regarding other SIRTs, lower serum SIRT-6 levels were found in patients with periodontitis compared to periodontally healthy subjects [44], and no differences in serum SIRT-3 and SIRT-4 levels were observed between patients with periodontitis and periodontally healthy subjects [45]. In addition, in a study by Tamaki et al. with rats, it was found that animals with periodontitis compared to rats without periodontitis showed lower expression of SIRT-1 in gingival tissues [46].

Taking into account that oxidation, inflammation and tissue degradation are involved in periodontitis [3,4,5,6,7], that SIRT-1 could modulate all these pathways [18,19,20,21,22,23,24,25,26,27,28,29], and that in the study by Tamaki Wu et, it was found that mice with periodontitis showed lower expression of SIRT-1 in gingival tissues [46], we hypothesize that SIRT-1 could play a protective role in human periodontitis.

The aims of our study were to compare salivary SIRT-1 concentrations in subjects with and without periodontitis in one study with a larger sample size to determine whether there exists an association between salivary SIRT-1 concentrations and the presence and severity of periodontitis and to analyze the potential capability of salivary SIRT-1 concentrations for periodontitis diagnosis. We determined SIRT-1 concentrations in saliva to be as non-invasive as possible in the sample taken.

## 2. Methods

### 2.1. Design and Subjects

The study protocol was approved by the Ethics Committee of Clinical Research of Hospital Universitario de Canarias (code: CHUC_2023_138; approval date: 30 November 2023), and each subject signed the informed consent form. This study has a cross-sectional design.

This study included two groups of participants, one with periodontitis (showing periodontal tissue loss) and another without periodontitis (showing localized gingivitis in less than 30% of sites or periodontal health). Periodontitis diagnosis and periodontitis severity classification were assessed using current internationally accepted criteria [3]. Subjects younger than 18 years old were excluded. The recruitment of subjects was carried out by the Clínica Dental Cándido (from La Laguna, Tenerife, Canary Islands, Spain).

### 2.2. Definitions

Periodontal health, define by bleeding, when probing was absent or only present in less than 10% of locations; clinical interproximal attachment loss was absent, and bone loss was absent.

Localized gingivitis with bleeding upon probing was observed in 10–30% of locations. Clinical interproximal attachment loss was absent, and bone loss was absent.

Periodontitis is defined as either having clinical interproximal attachment loss or bone loss. We used the following criteria to establish the severity of periodontitis: (1) Clinical loss of interproximal attachment: <3 mm (as stage I), 3–4 mm (as stage II) or ≥5 mm (as stages III or IV). (2) Radiographic loss of bone: coronal third < 15% (as stage I), coronal third of 15–33% (as stage II) or middle or apical third (as stages III or IV). (3) Loss of teeth: none (as stage I), 1 to 4 (as stage II) or ≥5 teeth (as stage III).

### 2.3. Variables Recorded

Consumption of drugs, tobacco, coffee, alcohol and tea was recorded. In addition, body mass index (BMI) (kg/m^2^), age, presence of obesity (whether BMI ≥ 30 kg/m^2^) and sex were recorded. Additionally, a personal history of arterial hypertension, hypercholesterolemia, cardiovascular disease, diabetes mellitus, oral cancer, rheumatoid arthritis and its need for treatment with methotrexate (at a dose for rheumatoid arthritis), or systemic lupus erythematosus was recorded. Moreover, we recorded the need for radiotherapy or immunosuppressive therapy, as well as dental hygiene.

### 2.4. Salivary Samples

We used the technique by Navazesh to obtain samples of whole non-stimulated saliva [47]. We collected saliva samples in the morning (8–10 A.M.) to minimize potential modifications by the circadian rhythm on salivary biomarkers. The subjects did not drink, smoke, or brush their teeth in the 2 h before sample collection. The participants rinsed their mouth three times with ten ml of deionized water. Then, they remain sat with their eyes open and head tilted forward for thirty minutes while trying to avoid orofacial movements. During this period, the subjects avoided swallowing saliva, allowing it to accumulate on the mouth before being collected in a bottle. Afterwards, saliva samples were centrifuged to remove cells and debris at 3000 rpm for 10 min. Then, the supernatant was pipetted and transferred into Eppendorf tubes. Finally, saliva samples were stored at −80 °C until the moment of determination.

Some participants were included in previous publications by our group, but in those publications, salivary concentrations of nitrites [48], malondialdehyde [49], uric acid [50] and 3-nitrotyrosine were determined [51]. However, the determination of salivary SIRT1 levels was the objective at this moment.

### 2.5. Salivary SIRT-1 Concentration Analysis

We used the Enzyme-Linked Immunosorbent Assay (ELISA) (Invitrogen, Thermo Fisher Scientific Inc., Whaltam, MA, USA) for the determination of salivary SIRT-1 concentrations. Briefly, 100 μL of the standard or 100 μL of saliva was deposited in each well and incubated overnight at 4–6 °C (the plate was covered with a plate sealer for incubation). Then, after washing each wall with a wash buffer, 100 μL of a solution containing a biotinylated detection antibody was added to each well, and the samples were incubated for 1 h at room temperature. Similarly, after washing each well with the wash buffer, 100 μL of a solution of Streptavidin linked to the Horseradish Peroxidase (HRP) enzyme was added into each well, and the samples were incubated for 45 min at room temperature. Then, after washing each well with the wash buffer, 100 μL of 3,3′,5,5′-tetramethylbenzidine (TMB) as the chromogenic reagent substrate was added into each well, and the samples were incubated for 35 min at room temperature while constantly avoiding direct light on the plate (the reagent is light-sensitive). Finally, we added 50 μL of the Stop Solution (1 M H_2_SO_4_) into each well at room temperature (and a yellow color was developed). The calibration curve from the kit ranged 0–300 ng/mL. We used a microplate spectrophotometer at 450 nm (Spectra MAX-190, Molecular Devices, Sunnyvale, CA, USA) to read samples and standards. The limit of detection was 0.087 ng/mL, with the intra-assay variation coefficient < 10% and the inter-assay variation coefficient < 12%.

### 2.6. Statistical Methods

We used the Mann–Whitney U-test and the chi-square test for the comparison between subjects without and with periodontitis as continuous variables (expressed in the form of median and percentiles (25–75)) and categorical variables (expressed in the form of the number of patients and a percentage), respectively. We explored the correlation between salivary SIRT-1 concentrations and periodontal stage using Spearman’s rho coefficient. We used Bonferroni correction due to multiple comparisons.

We carried out a multivariate logistic regression analysis to determine that variables were associated with periodontitis. In the regression analysis, those variables that showed a *p*-value ≤ 0.05 were included in the comparison between subjects without and with periodontitis, in addition to a reasonable number of patients associated with that event.

We performed a receiver operating characteristic (ROC) analysis to determine the capability of salivary SIRT-1 concentrations for periodontitis diagnosis, and we reported the area under curve (AUC). Negative and positive likelihood ratios, negative and positive predictive values, specificity and sensitivity for the cut-off of salivary SIRT1 concentrations < 1.2 ng/mL, and the Youden J index were used to select this cut-off [52].

We used SPSS version 17.0 (SPSS Inc., Chicago, IL, USA) and MedCalc Statistical Software version 22.016 (MedCalc Software Ltd., Ostend, Belgium) for statistical analyses.

## 3. Results

We included 85 subjects without periodontitis and 59 subjects with periodontitis. The number of subjects for each periodontal stage and its salivary SIRT-1 concentration are shown in Table 1, and significant differences in salivary SIRT-1 concentrations between subject groups were found (*p* < 0.001). In addition, we found a negative correlation between salivary 3-SIRT-1 concentrations and periodontitis severity (rho = −0.42; *p* < 0.001).

Periodontitis subjects with respect to non-periodontitis subjects had lower salivary SIRT-1 concentrations (*p* < 0.001). In addition, periodontitis subjects had higher rates of diabetes mellitus (*p* = 0.01), cardiovascular disease (*p* = 0.03), arterial hypertension (*p* = 0.001) and tobacco use (*p* < 0.001) and lower rates of tea consumption (*p* = 0.004), and they were older (*p* < 0.001). We found no statistically significant differences between subjects without and with periodontitis in terms of sex, coffee, alcohol, hypercholesterolemia, statins, body mass index, obesity, rheumatoid arthritis, methotrexate for rheumatoid arthritis, immunosuppressive therapy, and radiotherapy (Table 2). No subjects, either with or without periodontitis, had drug consumption, systemic lupus erythematosus, oral cancer, or metformin use.

Multiple logistic regression analysis showed that salivary SIRT-1 concentration < 1.2 ng/mL (OR = 2.84; 95% CI = 1.075–7.506; *p* = 0.04) and age (years) (OR = 1.11; 95% CI = 1.064–1.161; *p* < 0.001) were associated with periodontitis (Table 3).

**Figure 1:**
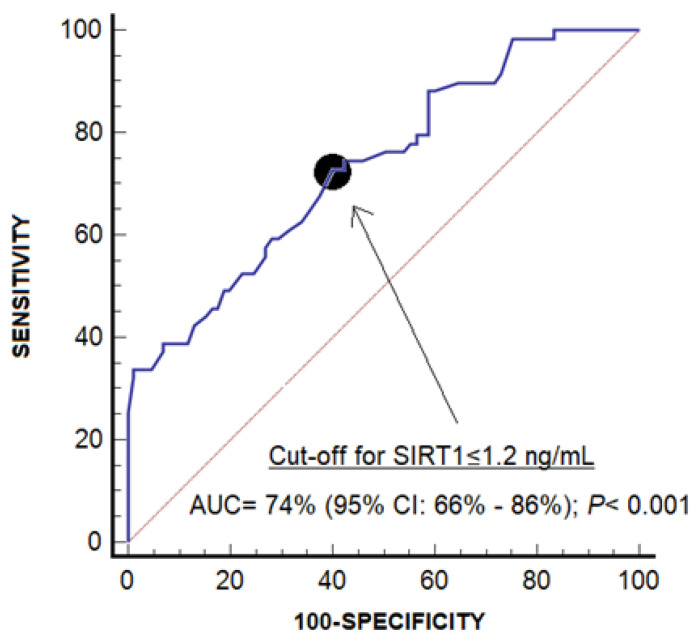
Receiver operating characteristic analysis to determine the capability of salivary sirtuin 1 (SIRT1) concentrations for periodontitis diagnosis.

Salivary SIRT-1 levels showed an AUC of 74% (95% CI = 66–86%; *p* < 0.001) for periodontitis diagnosis (Figure 1). The cut-off point selected for periodontitis diagnosis by salivary SIRT-1 levels < 1.2 ng/mL had a negative likelihood ratio of 0.5 (0.3–0.7), a positive likelihood ratio of 1.8 (1.4–2.5), a negative predictive value of 76% (67–83%), a positive predictive value of 56% (48–63%), a specificity of 60% (49–71%), and a sensitivity of 73% (60–84%).

We found an association between salivary SIRT-1 levels and age (rho = −0.27; *p* = 0.001). However, no significant associations were found between salivary SIRT-1 levels and body mass index (rho = 0.05; *p* = 0.55), coffee (*p* = 0.04), cardiovascular disease (*p* = 0.10), hypercholesterolemia (*p* = 0.95), sex (*p* = 0.70), obesity (*p* = 0.35), alcohol (*p* = 0.20), tea consumption (*p* = 0.68), tobacco use (*p* = 0.25), diabetes mellitus (*p* = 0.40), arterial hypertension (*p* = 0.08), rheumatoid arthritis (*p* = 0.53), metrotexate (*p* = 0.61), immunosuppressive therapy (*p* = 0.61), radiotherapy (*p* = 0.06) and statins (*p* = 0.95) after Bonferroni correction for multiple comparisons.

## 4. Discussion

Our study had the highest sample size reporting salivary SIRT-1 concentrations in periodontitis. We found lower salivary SIRT-1 concentrations in subjects with periodontitis than those without it. We found an association between salivary SIRT-1 concentrations with periodontitis and its severity. We also found that salivary SIRT-1 concentrations may play a role in the diagnosis of periodontitis.

Salivary SIRT-1 concentrations in periodontitis patients have been scarcely studied, and no differences were found in salivary SIRT-1 concentrations between periodontitis patients and periodontally healthy subjects in any of those studies [36,37,38]. In the study by Kluknavská et al. with 40 periodontitis patients and 43 periodontally healthy subjects, higher salivary SIRT-2 levels were found in periodontitis patients than in healthy subjects, and no differences were found in salivary SIRT-1 levels [36]. In the study by Sayedyousef et al. with 36 periodontitis patients and 30 periodontally healthy subjects, lower serum SIRT-1 levels were found in periodontitis patients than in periodontally healthy subjects, and no differences were found in salivary SIRT-1 levels [37]. In the study by İyigün et al. with 32 periodontitis patients and 17 periodontally healthy subjects, no differences were found in salivary SIRT-1 levels between periodontitis and periodontally healthy subjects [38].

SIRT-1 concentrations in biological samples other than saliva in patients with periodontitis have been sparsely studied, and the results are contradictory [39,40,41,42,43]. In the study by Kriaučiūnas et al., which included 201 patients with periodontitis and 500 periodontally healthy subjects, no differences were found in serum SIRT-1 levels between patients with periodontitis and periodontally healthy subjects [39]. In the study by Caribé et al. with 40 periodontitis patients and 38 periodontally healthy subjects, higher serum SIRT-1 levels were found in periodontitis patients than in periodontally healthy subjects [40]. In the study by Purohit et al. with 70 periodontitis patients (35 smokers and 35 non-smokers) and 35 periodontally healthy subjects, lower serum SIRT-1 levels were found in periodontitis patients who smoke than in non-smoking periodontitis patients and periodontally healthy subjects, and no differences were found in serum SIRT-1 levels between non-smoking periodontitis patient and periodontally healthy subjects [41]. In the study by Kuo et al. with 33 periodontitis patients and 11 periodontally healthy subjects, higher expression of SIRT-1 was found in the gingival tissue samples of periodontitis patients than in those of periodontally healthy subjects [42]. In the study by Yang et al., which included 213 patients with periodontitis and 95 periodontally healthy subjects, lower SIRT-1 expression was found in the gingival tissue samples from patients with periodontitis than in those from periodontally healthy subjects [43]. Regarding other SIRTs, lower serum SIRT-6 levels were found in patients with periodontitis compared to periodontally healthy subjects [44], and no differences were observed in serum SIRT-3 and SIRT-4 levels between patients with periodontitis and periodontally healthy subjects [45].

Therefore, our study has a larger sample size (144 subjects) than other previously published studies about salivary SIRT-1 concentrations in periodontitis (the highest with 83 subjects in the study by Kluknavská [36]). In addition, considering the results of previous studies, a novel finding of our study was that salivary SIRT-1 concentrations were lower in subjects with periodontitis than those without periodontitis. Our findings could be in consonance with those of a previous study by Tamaki et al. with rats, which found that animals with periodontitis compared to those without periodontitis showed lower expression of SIRT-1 in gingival tissues [46].

Another novel finding of our study was that there is an association between low salivary SIRT-1 concentrations and the presence of periodontitis independent of other associated factors according to the regression analysis. Several risk factors for periodontitis have been reported, including tea, tobacco, alcohol, and coffee consumption; immunosuppression; oral cancer; dental hygiene; consumption of drugs [4]; age [8]; obesity [9]; rheumatoid arthritis [10]; arterial hypertension [11]; diabetes mellitus [12]; systemic lupus erythematosus [13]; sex [14]; statins [15]; metformin [16]; socioeconomic status; and diet [17]. We found that age was another factor associated with periodontitis, as previously reported [8]; however, no other variables were found to be associated with periodontitis. In addition, given the cross-sectional nature of the present study and the absence of a temporal sequence, our findings do not allow for causal inferences. Therefore, salivary SIRT-1 levels should not be interpreted as a risk factor for periodontitis but rather as an associated factor. The observed association suggests a potential link between SIRT-1 and periodontitis; nevertheless, establishing a causal relationship would require longitudinal designs or specific causal analyses.

We found that salivary SIRT-1 concentrations have the capacity to help in the diagnosis of periodontitis. However, the area under the curve of salivary SIRT-1 concentrations and the statistical parameters for the selected cut-off of salivary SIRT-1 concentrations were not high [53]. Therefore, salivary SIRT-1 concentrations could help in the diagnosis of periodontitis, but they could not be used alone as the sole diagnostic parameter. Further investigations are necessary to confirm these preliminary findings.

In some previous studies, a negative association between serum SIRT-1 levels and periodontitis severity was found [37,41]. Thus, considering the results of those previous studies, another novel finding of our study was that there is an association between low salivary SIRT-1 concentrations and the severity of periodontitis. Our findings could be in consonance with those of previous studies on serum SIRT-1 levels.

An interesting point is that SIRT concentrations could be modulated by different agents. Different SIRT activators and SIRT inhibitors have been explored [30,31,32,33,34,35]. Among the SIRT inhibitors are selisistat (also known as Ex-527 or SEN0014196), cambinol, AGK2, SirReal2, ELT-11c, compound 1, compound 8, compound 15e, compound 28e and compound 39. The SIRT activators include natural extracts (resveratrol and piceatannol) and synthetic drugs (SRT1460, SRT1720, SRT2104, SRT2183, SRT2379, SRT3025, and UBCS039). Resveratrol is the first and most studied SIRT activator. Resveratrol is a natural antioxidant found in foods such as the skin of red grapes, peanuts, blackberries, blueberries, raspberries, dark chocolate, walnuts, hazelnuts and almonds.

In addition, the use of resveratrol, specifically for periodontitis, in rat models has been associated with higher SIRT-1 expression, higher antioxidants levels and lower oxidation biomarkers levels in gingival tissues, and lower bone loss [46,54,55,56]. In the study by Tamaki et al., they found that rats with periodontitis receiving resveratrol showed increased expression of SIRT-1 and of Nrf2, decreased levels of 8-hydroxydeoxyguanosine, decreased levels of nitrotyrosine, decreased levels of proinflammatory cytokines and relieved alveolar bone resorption [46]. In the study by Cirano et al., it was found that rats with periodontitis receiving resveratrol showed higher expression of SIRT-1 in gingival tissues, lower IL-1 and IL-6 concentrations, higher SOD concentrations and lower bone loss [54]. In the study by Correa et al., it was found that rats with periodontitis receiving resveratrol showed higher expression of SIRT-1 in gingival tissues, higher levels of SOD, and lower bone loss [55]. In the study by Zhu et al., it was found that rats with periodontitis receiving manganese dioxide coupled with a metal–organic framework showed higher expression of SIRT-1 in gingival tissues, higher levels of SOD and catalase, and alleviated bone resorption [56].

We must declare different limitations of our study. For example, the determination of SIRT-1 concentrations in other samples was not carried (such as in blood, gingival tissue or gingival crevicular fluid), but we wanted to be as non-invasive as possible in conducting our study. In addition, recruitment was carried out at a single clinic; therefore, the results cannot be generalized to the overall population. With a larger sample size, additional factors associated with periodontitis might have been identified. Furthermore, we did not register other confounding factors, such as socioeconomic status, diet, periodontal treatment history, routine dental examinations or oral prophylaxis, and plaque scores were not addressed. Moreover, the observational nature of this study could imply the presence of potential bias, and unmeasured variables may have influenced the observed findings. In addition, the sample size was not calculated; however, it was sufficient to find an association between low salivary TRX-1 levels and periodontitis. Furthermore, although the examination of periodontal status was carried out by qualified periodontists, inter-examiner agreement was not evaluated. In addition, freeze–thaw cycles and long-term storage at −80 °C could have influenced the salivary concentrations of the biomarkers. Lastly, we have not matched the subject groups and did not include other cohorts to validate the findings.

However, despite the limitations of our study, we think that the strengths of our study are that it has the largest sample size of the studies published so far on salivary SIRT-1 concentration in periodontitis and that it is the first study that reports the association between low salivary SIRT-1 levels and periodontitis presence and severity. We found an association between salivary SIRT-1 levels and age; however, no other significant associations were found between salivary SIRT-1 levels and the other variables registered. In addition, multiple logistic regression analysis showed that the variables associated independently with periodontitis were salivary SIRT-1 concentrations and age. Therefore, further investigations are necessary to confirm these preliminary findings.

Finally, with respect to future research directions, it could be interesting to determine the role of salivary TRX-1 concentrations in the development of periodontitis, periodontitis severity and periodontitis diagnosis in larger studies controlling for more variables and to determine the effects of the administration of agents that modulate thioredoxin system activity in randomized clinical trials on the clinical evolution of periodontitis and on the concentrations of oxidative and inflammatory buccal biomarkers. We think that the findings of our study could motivate research about salivary SIRT-1 levels in periodontitis. Traditionally, the diagnosis and monitoring of the evolution of periodontitis have been based on probing and radiographic assessments. However, different factors related to the examiner (experience or pressure applied during periodontal probing) or the patient (limited mouth opening) could affect the evaluation of periodontitis presence and severity [57,58,59,60,61,62]. Thus, the determination of salivary biomarker concentrations (for example SIRT-1) could help those responsible for the diagnosis and evolution monitor of periodontitis.

## 5. Conclusions

Our study had the highest sample size reporting salivary SIRT-1 concentrations in periodontitis. We found lower salivary SIRT-1 concentrations in subjects with periodontitis than in those without periodontitis, and this finding differs from previous studies reporting no differences between patients with periodontitis and periodontally healthy subjects. We found a negative association between salivary SIRT-1 concentrations and age. We found that the variables associated with periodontitis were low salivary SIRT-1 concentrations and age. We also found an association between salivary SIRT-1 concentrations and periodontitis severity (lower levels to higher severity). We found that salivary SIRT-1 concentrations may play a role in the diagnosis of periodontitis as an adjunct to other markers; however, they could not be used alone as the sole diagnostic parameter because their diagnostic capability was not high.

However, our study had several limitations, as SIRT-1 concentrations were not determined in other biological samples. This study was unicentric; the sample size was relatively limited to identify other factors associated with periodontitis; some potential variables associated with periodontitis were not registered, and there was a possible presence of bias due to the observational design of this study. Inter-examiner agreement of periodontal status was not evaluated, and long-term −80 °C storage could have influenced the concentrations of salivary biomarkers. Therefore, further investigations are necessary to confirm these preliminary findings.

## Figures and Tables

**Table 1:** Number of subjects and salivary sirtuin 1 concentration in each periodontal stage.

	Total (*n* = 144)	Salivary Sirtuin-1 (ng/mL) Median (p 25–75)	*p*-Value
			<0.001
Periodontal health-n (%)	85 (59.0)	1.26 (1.08–1.63)	
Periodontitis stage I-n (%)	17 (11.8)	1.15 (0.90–1.37)	
Periodontitis stage II-n (%)	23 (16.0)	1.11 (0.88–1.39)	
Periodontitis stage III-n (%)	13 (9.0)	1.03 (0.84–1.12)	
Periodontitis stage IV-n (%)	6 (4.2)	0.91 (0.81–1.06)	

**Table 2:** Comparisons between subjects without and with periodontitis.

	Subjects Without Periodontitis (*n* = 85)	Subjects with Periodontitis (*n* = 59)	*p*-Value
Gender female—n (%)	62 (72.9)	36 (61.0)	0.15
Age (years)—median (p 25–75)	36 (20–45)	59 (51–68)	<0.001
Arterial hypertension—n (%)	4 (4.7)	18 (30.5)	0.001
Cardiovascular disease—n (%)	0	4 (6.8)	0.03
Hypercholesterolemia—n (%)	2 (2.4)	2 (3.4)	0.54
Statins—n (%)	2 (2.4)	2 (3.4)	0.54
Diabetes mellitus—n (%)	0	5 (8.5)	0.01
Rheumatoid arthritis—n (%)	2 (2.4)	3 (5.1)	0.40
Metrotexate for rheumatoid arthritis—n (%)	2 (2.4)	0	0.51
Immunosuppressive therapy—n (%)	2 (2.4)	1 (1.7)	0.99
Radiotherapy—n (%)	0	1 (1.7)	0.41
Body mass index (kg/m^2^)—median (p 25–75)	24.5 (22.4–26.6)	24.8 (22.5–27.7)	0.52
Obesity-n (%)	11 (12.9)	8 (13.6)	0.99
Tobacco			<0.001
Non-smoker—n (%)	66 (77.6)	25 (42.4)	
Ex-smoker—n (%)	9 (10.6)	20 (33.9)	
Smoker—n (%)	10 (11.8)	14 (23.7)	
Coffee—n (%)	68 (80.0)	52 (88.1)	0.26
Tea—n (%)	19 (22.4)	3 (5.1)	0.004
Alcohol—n (%)	33 (38.8)	31 (52.5)	0.13
Salivary sirtuin-1 levels (ng/mL)—median (p 25–75)	1.26 (1.08–1.63)	1.05 (0.87–1.26)	<0.001
Salivary sirtuin-1 levels < 1.2 ng/mL-n (%)	34 (40.0)	43 (72.9)	<0.001

**Table 3:** Multiple logistic regression analysis to determine that factors are associated with periodontitis.

	Odds Ratio	95% Confidence Interval	*p*-Value
Age (years)	1.11	1.064–1.161	<0.001
Salivary sirtuin-1 levels < 1.2 ng/mL (yes vs. no)	2.84	1.075–7.506	0.04
Tobacco (never smoker, ex-smoker, smoker)	---	---	0.17
Arterial hypertension (yes vs. no)	1.23	0.287–5.228	0.78
Tea consumption (yes vs. no)	0.27	0.042–1.665	0.16

## Data Availability

The data that supports the findings of this study are available from the corresponding author upon reasonable request.

## Abbreviation

SIRT-1	sirtuin-1
